# From theories of aging to anti-aging interventions

**DOI:** 10.3389/fgene.2014.00276

**Published:** 2014-08-14

**Authors:** Alexey A. Moskalev, Elena G. Pasyukova

**Affiliations:** ^1^Radiation Ecology, Laboratory of Molecular Radiobiology and Gerontology, Institute of Biology of Komi Science Center of Ural Branch of RASSyktyvkar, Russia; ^2^Syktyvkar State UniversitySyktyvkar, Russia; ^3^Moscow Institute of Physics and Technology (State University)Dolgoprudny, Russia; ^4^Laboratory of Genome Variation, Institute of Molecular Genetics, Russian Academy of SciencesMoscow, Russia

**Keywords:** aging, evolution, genetics, epigenetics, geroprotectors

The 2nd International Conference «Genetics of aging and longevity » took place 22–25 April, 2012 in Moscow, Russia. Top gerontologists and geneticists from 25 countries around the world discussed the current problems in many areas related to the genetics of longevity and mechanisms of aging. Following this meeting, a collection of articles based on the talks, reports and experimental outcomes related to the topics of the conference was published in Frontiers in Genetics of Aging. This collection represents a comprehensive prospect of recent advances in genetics of aging, ranging from theoretical questions to practical approaches aimed to delay aging and prolong lifespan.

Unraveling the proximal cause of aging and creating a universal aging theory remains a difficult and ambitious task. Complicated mechanisms that preserve homeostasis and prolong lifespan involve interactions of numerous genes, metabolic pathways and environmental cues. Genetic and environmental variations, combinations of programmed and random events underlying the aging process further complicate the objective. Trindade et al. (2013) emphasize that a universal evolutionary aging theory must reconcile “trade-offs (pleiotropy or hitchhiking effect), retrogression (mutational load and drift) and direct adaptation (“program”).”

The important notion that aging and lifespan are intimately related to various life history traits is highlighted in several articles from the collection. Novoseltsev and Novoseltseva (2013) used mathematical approaches to assess the origin of stochasticity in Drosophila reproduction, in relation to life expectancy. To add to mathematical predictions, analysis of long-term captive breeding of water voles demonstrated that individuals who began breeding at an older age had a significantly longer lifespan and produced more offspring (Nazarova, 2013).

These results resonate with the basis of the aging theory that set the grounds for a heated debate concerning the proximal cause of aging (Zimniak, 2012). In spite of many new insights into regulatory mechanisms that affect the aging process, the author remains true to the hypothesis of molecular damage and argues that hyper function proposed as a universal cause of aging by Blagosklonny (for references, see Zimniak, 2012) is only one of several sources of molecular damage. It is not proved, however, that a single proximal cause of aging does exist. According to the point of view of Lehmann et al. (2013), telomere length and body temperature are independent drivers of mammalian longevity.

New facts are needed to advance our understanding of aging, and studies of model organisms continue to provide us with valuable information. There are five articles in the collection, which represent new discoveries in genetics of aging of *Caenorhabditis elegans* (Bharill et al., 2013; Coburn and Gems, 2013), *Drosophila melanogaster* (Rogina and Helfand, 2013; Shostal and Moskalev, 2013), and mice (Bartke and Westbrook, 2012). In one of the most intriguing articles, Coburn and Gems (2013) come to the conclusion that the blue fluorescence of gut granules, a marker of death in *C. elegans*, is issued from anthranilic acid glucosyl esters, rather than from lipofuscin. They believe that this removes one reason for believing that worm aging is caused by accumulation of molecular damage, thus entering into the extramural discussion with Zimniak (2012).

In recent years, studies of long-lived wild animals, mainly rodents have complemented studies of model species and open new perspectives in aging research. In particular, the longevity of these species is correlated with cancer resistance, and analysis of molecular mechanisms underlying this property can benefit human health if such mechanisms can be activated in human cells (Azpurua and Seluanov, 2013).

Analysis of human populations represents an invaluable source of information concerning genetic and environmental factors promoting a long and healthy life. The results of several longitudinal studies of centenarians suggest, on the one hand, that factors responsible for exceptional longevity and health are not necessary the same and centenarians often experience chronic age-related diseases, but are able to cope with them. On the other hand, these results suggest that postponing aging changes is associated with extreme longevity (Sebastiani and Perls, 2012; Yashin et al., 2013). To add to the complexity, it is likely that the genetic component of extreme longevity includes many genes with modest effects (Sebastiani and Perls, 2012), which underscores once again that there is no simple universal recipe for a long life.

Unraveling fundamentals of exceptional longevity is essential for the concept of aging; for practical reasons, it may be even more important to get at the mechanisms linking lifespan and health. Theoretical aspects of this problem are reviewed in two articles providing compact but thorough description of the role of mitophagy (Palikaras and Tavernarakis, 2012) and Alu elements (Mustafina, 2013) in aging, while Djansugurova et al. (2013) present new data on genetic markers of cancers.

Understanding the molecular mechanisms underlying aging and age-associated diseases could bring us closer to the development of novel, efficient, anti-aging treatments. In this regard, one of the latest trends, the use of stem cells to increase lifespan, is described by Kovina et al. (2013).

Recently, there is also a great hope for the development of target-specific drugs for age-associated chronic diseases and, possibly, anti-aging drugs. Several articles in this collection are related to this problem. Vaiserman and Pasyukova (2012) present arguments that the development of specific drugs which target epigenetic pathways could be a highly promising anti-aging strategy. Bharill and co-authors demonstrated that commercially available inhibitors of AKT/FOXO signaling are able to enhance longevity and tolerance to oxidative stress in *C. elegans* (Bharill et al., 2013). However, the extreme complexity of the genetic control of homeostasis and aging predetermines caution in the use of new drugs. An example of possible side effects of medical treatments is proposed by Smith et al. (2013): the premature and accelerated aging of HIV-patients can be caused by adverse effects of antiretroviral drugs, specifically those that cause severe mitochondrial damage.

Another problem in drug discovery is the difficulty of extrapolating of the results from model species to humans and the time it takes to evaluate the effects of various interventions on longevity in humans. This problem is addressed in the article of Zhavoronkov et al. (2014) who propose a method for screening and ranking the possible geroprotectors before conducting pre-clinical work and expensive clinical trials.

## Conflict of interest statement

The authors declare that the research was conducted in the absence of any commercial or financial relationships that could be construed as a potential conflict of interest.
